# Supplementary figures and images for: Non-significant influence between aerobic and anaerobic sample transport materials on gut (fecal) microbiota in healthy and fat-metabolic disorder Thai adults
Source: PeerJ. 2024 Apr 19;12:e17270. doi: 10.7717/peerj.17270 (PMC11034497; doi:10.7717/peerj.17270)

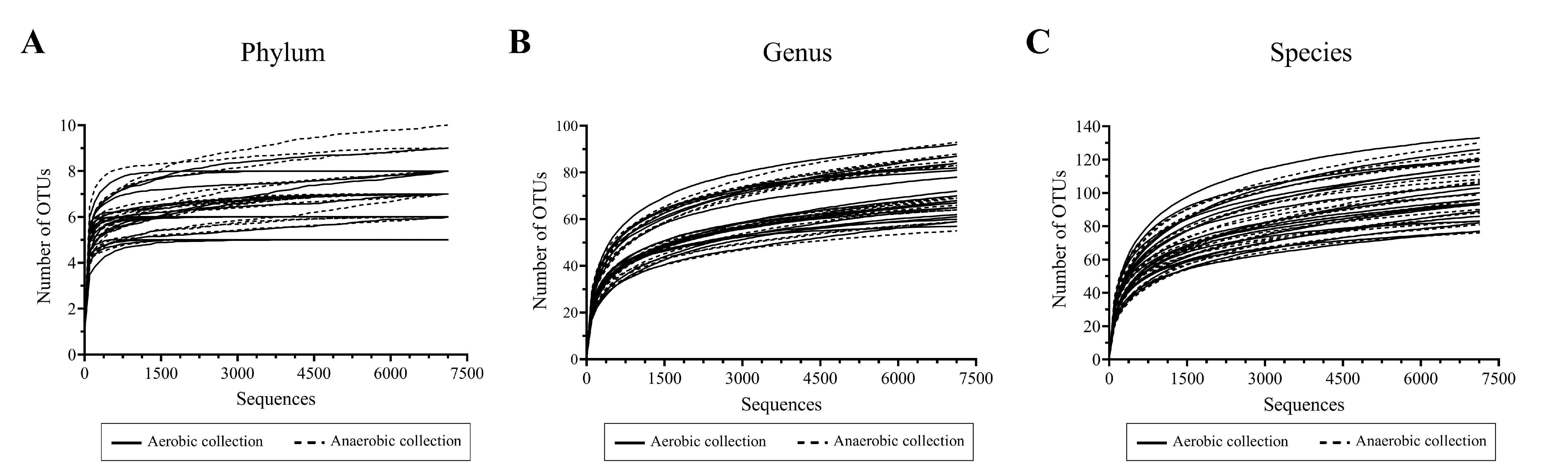

Supplement: Supplemental Information 1 [file peerj-12-17270-s001.jpg]

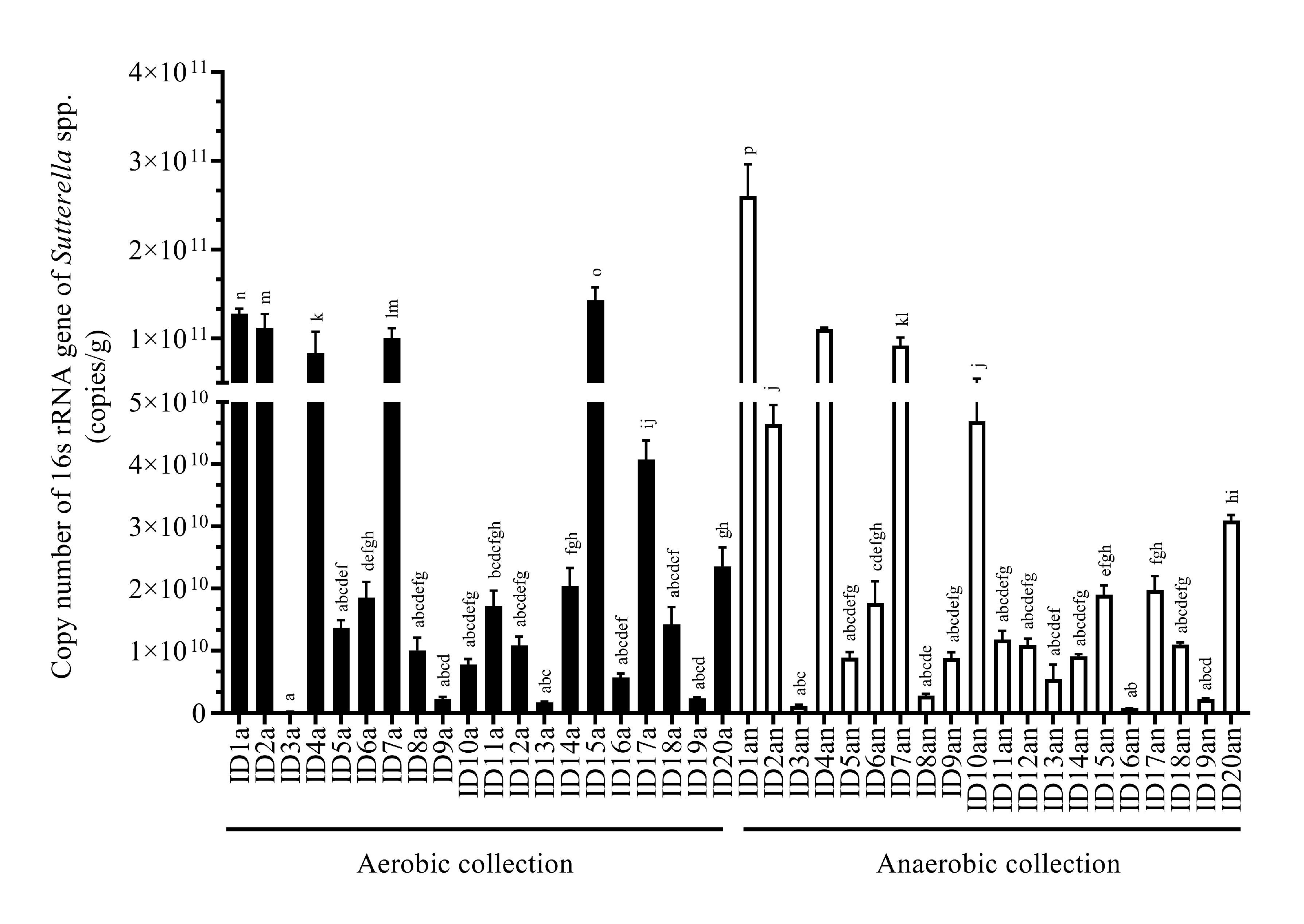

Supplement: Supplemental Information 2 [file peerj-12-17270-s002.jpg]

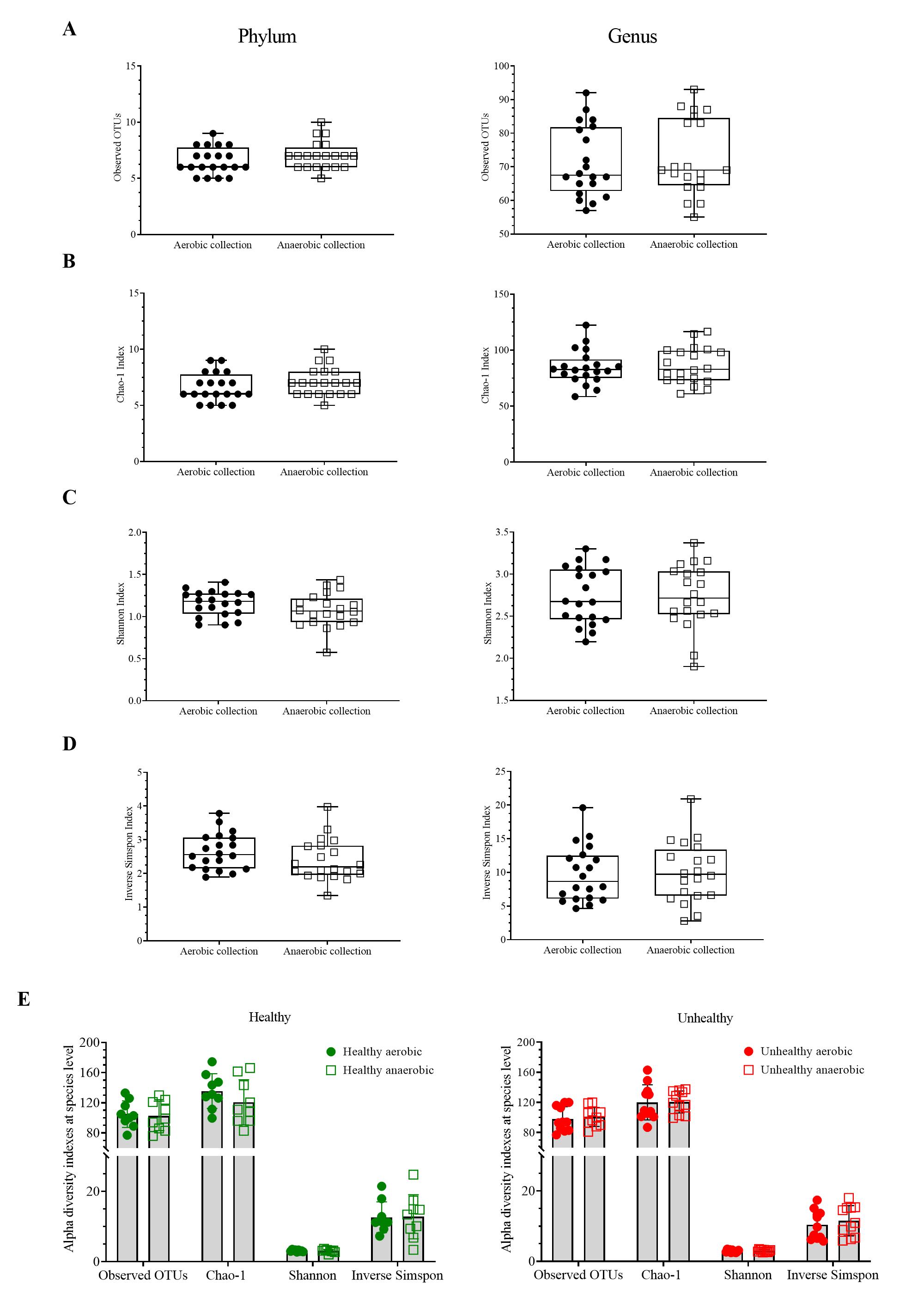

Supplement: Supplemental Information 3 — Statistical differences between groups were tested using Student’s t-test (P < 0.05), and no statistical difference was found: P > 0.05. [file peerj-12-17270-s003.jpg]

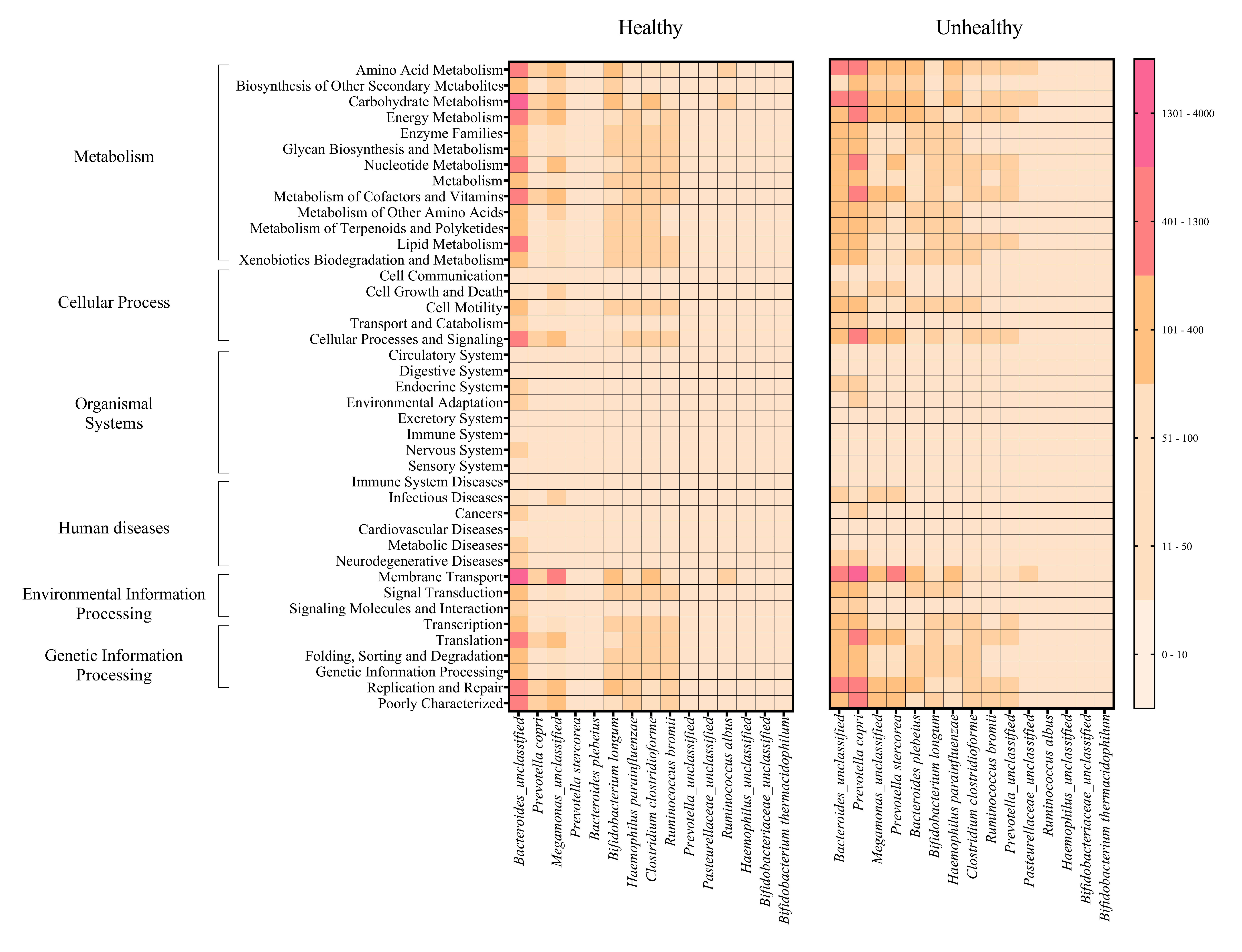

Supplement: Supplemental Information 4 — Microbial metabolic functions were estimated according to KEGG pathways, and a different color from light nude to pink represents the level of quantitative microbial metabolic function abundance from absence to the highest presence level (scale in vertical bar chart). [file peerj-12-17270-s004.jpg]
