# Supplementary material for: Non-significant influence between aerobic and anaerobic sample transport materials on gut (fecal) microbiota in healthy and fat-metabolic disorder Thai adults
Source: PeerJ. 2024 Apr 19;12:e17270. doi: 10.7717/peerj.17270 (PMC11034497; doi:10.7717/peerj.17270)
Supplement: Supplemental Information 5 [file peerj-12-17270-s005.docx]

**Table S1.** Number of raw reads, quality reads, OTUs and Good’s coverage (%) at phylum, genus and species level of (A) aerobic sample transport and (B) anaerobic sample transport groups.

**A**

| **Sample IDs** | **Number of reads** | | **Phylum** | | **Genus** | | **Species** | |
| --- | --- | --- | --- | --- | --- | --- | --- | --- |
|  | **Raw** | **Quality** | **OTUs** | **Good's coverage** | **OTUs** | **Good's coverage** | **OTUs** | **Good's coverage** |
| **ID1a** | 118505 | 94439 | 5 | 100.00 | 65 | 99.80 | 82 | 99.73 |
| **ID2a** | 80082 | 60357 | 6 | 100.00 | 57 | 99.94 | 83 | 99.87 |
| **ID3a** | 71065 | 48416 | 7 | 99.99 | 70 | 99.80 | 93 | 99.71 |
| **ID4a** | 104163 | 78155 | 6 | 100.00 | 59 | 99.78 | 77 | 99.73 |
| **ID5a** | 81255 | 58685 | 5 | 100.00 | 60 | 99.90 | 77 | 99.80 |
| **ID6a** | 97153 | 67858 | 8 | 100.00 | 87 | 99.75 | 116 | 99.62 |
| **ID7a** | 89970 | 59716 | 7 | 100.00 | 84 | 99.78 | 113 | 99.68 |
| **ID8a** | 79423 | 60230 | 5 | 100.00 | 62 | 99.85 | 86 | 99.75 |
| **ID9a** | 84451 | 54372 | 7 | 100.00 | 81 | 99.87 | 126 | 99.61 |
| **ID10a** | 91965 | 65186 | 6 | 100.00 | 67 | 99.83 | 96 | 99.73 |
| **ID11a** | 84075 | 51981 | 8 | 99.97 | 82 | 99.90 | 120 | 99.71 |
| **ID12a** | 108682 | 71907 | 8 | 99.99 | 84 | 99.80 | 120 | 99.76 |
| **ID13a** | 88285 | 64335 | 6 | 100.00 | 61 | 99.83 | 100 | 99.68 |
| **ID14a** | 26181 | 16724 | 5 | 100.00 | 67 | 99.85 | 105 | 99.71 |
| **ID15a** | 20825 | 14460 | 6 | 100.00 | 65 | 99.85 | 94 | 99.66 |
| **ID16a** | 120845 | 87511 | 6 | 100.00 | 67 | 99.80 | 89 | 99.62 |
| **ID17a** | 84156 | 54143 | 6 | 99.99 | 68 | 99.79 | 103 | 99.64 |
| **ID18a** | 22177 | 13904 | 7 | 100.00 | 92 | 99.80 | 133 | 99.73 |
| **ID19a** | 36802 | 23328 | 9 | 99.99 | 72 | 99.78 | 94 | 99.75 |
| **ID20a** | 27583 | 16628 | 8 | 100.00 | 78 | 99.80 | 116 | 99.66 |

**B**

| **Sample IDs** | **Number of reads** | | **Number of reads** | | **Genus** | | **Species** | |
| --- | --- | --- | --- | --- | --- | --- | --- | --- |
|  | **Raw** | **Quality** | **OTUs** | **Good's coverage** | **OTUs** | **Good's coverage** | **OTUs** | **Good's coverage** |
| **ID1an** | 22743 | 16527 | 6 | 100.00 | 69 | 99.86 | 88 | 99.73 |
| **ID2an** | 25254 | 15573 | 7 | 100.00 | 67 | 99.85 | 93 | 99.72 |
| **ID3an** | 11463 | 7137 | 6 | 100.00 | 64 | 99.87 | 90 | 99.79 |
| **ID4an** | 32891 | 21548 | 6 | 100.00 | 55 | 99.87 | 81 | 99.75 |
| **ID5an** | 34344 | 24527 | 6 | 100.00 | 66 | 99.83 | 86 | 99.78 |
| **ID6an** | 50952 | 33129 | 9 | 100.00 | 93 | 99.73 | 130 | 99.58 |
| **ID7an** | 119967 | 78021 | 7 | 100.00 | 83 | 99.78 | 108 | 99.79 |
| **ID8an** | 60443 | 42347 | 7 | 99.97 | 69 | 99.79 | 107 | 99.57 |
| **ID9an** | 67837 | 40939 | 7 | 100.00 | 85 | 99.79 | 121 | 99.64 |
| **ID10an** | 43606 | 30408 | 7 | 100.00 | 68 | 99.78 | 99 | 99.80 |
| **ID11an** | 48478 | 31043 | 7 | 99.99 | 88 | 99.73 | 119 | 99.72 |
| **ID12an** | 48462 | 29440 | 9 | 99.99 | 87 | 99.78 | 120 | 99.79 |
| **ID13an** | 21089 | 12679 | 8 | 99.99 | 59 | 99.82 | 83 | 99.80 |
| **ID14an** | 21849 | 12940 | 6 | 99.99 | 68 | 99.85 | 111 | 99.75 |
| **ID15an** | 29929 | 18629 | 7 | 100.00 | 64 | 99.87 | 96 | 99.62 |
| **ID16an** | 38978 | 29505 | 6 | 100.00 | 59 | 99.86 | 76 | 99.85 |
| **ID17an** | 64451 | 46372 | 5 | 100.00 | 70 | 99.75 | 94 | 99.75 |
| **ID18an** | 63622 | 44519 | 7 | 100.00 | 87 | 99.79 | 124 | 99.68 |
| **ID19an** | 22446 | 13066 | 8 | 99.99 | 70 | 99.76 | 100 | 99.65 |
| **ID20an** | 19512 | 12833 | 10 | 99.99 | 83 | 99.83 | 106 | 99.72 |
