# Supplementary material for: Non-significant influence between aerobic and anaerobic sample transport materials on gut (fecal) microbiota in healthy and fat-metabolic disorder Thai adults
Source: PeerJ. 2024 Apr 19;12:e17270. doi: 10.7717/peerj.17270 (PMC11034497; doi:10.7717/peerj.17270)
Supplement: Supplemental Information 6 — Only phylum (or genus, or species) OTU with > 1% average abundance was displayed. The k_ abbreviated kingdom bacteria but unclassified phylum; p, phylum; o_, order; f_, family; g_, genus; and s_, species. Statistical test was compared for each OTU between groups and no statistical P < 0.05 was found. [file peerj-12-17270-s006.docx]

**Table S2.** Average percent microbiota compositions at phylum, genus and species levels, and statistical comparison of each OTU between aerobic and anaerobic sample transport groups.

Only phylum (or genus, or species) OTU with > 1% average abundance was displayed. The k_ abbreviated kingdom bacteria but unclassified phylum; p, phylum; o_, order; f_, family; g_, genus; and s_, species. Statistical test was compared for each OTU between groups and no statistical P < 0.05 was found.

| **OTUs** | | **Groups** | **N** | **Mean** | **SE** | **P-value** |
| --- | --- | --- | --- | --- | --- | --- |
| **Phylum level** | k__Bacteria_unclassified | aerobic | 20 | 1.54 | 0.44 | 0.973 |
|  |  | anaerobic | 20 | 1.52 | 0.53 |  |
|  | p__Firmicutes | aerobic | 20 | 48.58 | 3.83 | 0.210 |
|  |  | anaerobic | 20 | 55.49 | 3.84 |  |
|  | p__Bacteroidetes | aerobic | 20 | 25.44 | 2.92 | 0.622 |
|  |  | anaerobic | 20 | 23.20 | 3.43 |  |
|  | p__Proteobacteria | aerobic | 20 | 16.39 | 2.29 | 0.134 |
|  |  | anaerobic | 20 | 11.66 | 2.07 |  |
|  | p__Actinobacteria | aerobic | 20 | 5.45 | 1.28 | 0.815 |
|  |  | anaerobic | 20 | 5.89 | 1.32 |  |
|  | p__Fusobacteria | aerobic | 20 | 2.39 | 1.20 | 0.763 |
|  |  | anaerobic | 20 | 1.93 | 0.94 |  |
| **Genus level** | g__Bifidobacterium | aerobic | 20 | 2.60 | 0.73 | 0.665 |
|  |  | anaerobic | 20 | 3.16 | 1.06 |  |
|  | g__Collinsella | aerobic | 20 | 2.40 | 1.02 | 0.864 |
|  |  | anaerobic | 20 | 2.20 | 0.52 |  |
|  | g__Bacteroides | aerobic | 20 | 13.17 | 2.37 | 0.758 |
|  |  | anaerobic | 20 | 12.12 | 2.39 |  |
|  | g__Prevotella | aerobic | 20 | 8.06 | 3.02 | 0.744 |
|  |  | anaerobic | 20 | 6.65 | 3.04 |  |
|  | g__Alistipes | aerobic | 20 | 1.60 | 0.41 | 0.959 |
|  |  | anaerobic | 20 | 1.57 | 0.42 |  |
|  | f__Lachnospiraceae_unclassified | aerobic | 20 | 4.30 | 0.57 | 0.178 |
|  |  | anaerobic | 20 | 5.44 | 0.60 |  |
|  | g__Blautia | aerobic | 20 | 2.52 | 0.72 | 0.450 |
|  |  | anaerobic | 20 | 3.27 | 0.66 |  |
|  | g__Coprococcus | aerobic | 20 | 1.70 | 0.30 | 0.213 |
|  |  | anaerobic | 20 | 2.43 | 0.49 |  |
|  | g__Lachnospira | aerobic | 20 | 1.97 | 0.48 | 0.537 |
|  |  | anaerobic | 20 | 2.69 | 1.06 |  |
|  | g__Roseburia | aerobic | 20 | 3.73 | 0.63 | 0.339 |
|  |  | anaerobic | 20 | 4.87 | 1.00 |  |
|  | f__Ruminococcaceae_unclassified | aerobic | 20 | 3.28 | 0.74 | 0.985 |
|  |  | anaerobic | 20 | 3.30 | 0.81 |  |
|  | g__Faecalibacterium | aerobic | 20 | 11.35 | 1.40 | 0.546 |
|  |  | anaerobic | 20 | 12.58 | 1.45 |  |
|  | g__Oscillospira | aerobic | 20 | 4.18 | 0.92 | 0.962 |
|  |  | anaerobic | 20 | 4.24 | 0.93 |  |
|  | g__Ruminococcus | aerobic | 20 | 3.11 | 0.49 | 0.997 |
|  |  | anaerobic | 20 | 3.11 | 0.58 |  |
|  | g__Megamonas | aerobic | 20 | 3.34 | 1.79 | 0.697 |
|  |  | anaerobic | 20 | 4.64 | 2.81 |  |
|  | g__Phascolarctobacterium | aerobic | 20 | 1.98 | 1.00 | 0.482 |
|  |  | anaerobic | 20 | 1.22 | 0.41 |  |
|  | o__Clostridiales_unclassified | aerobic | 20 | 1.40 | 0.35 | 0.962 |
|  |  | anaerobic | 20 | 1.42 | 0.40 |  |
|  | g__Fusobacterium | aerobic | 20 | 2.33 | 1.17 | 0.762 |
|  |  | anaerobic | 20 | 1.88 | 0.92 |  |
|  | g__Sutterella | aerobic | 20 | 9.38 | 2.14 | 0.313 |
|  |  | anaerobic | 20 | 6.48 | 1.87 |  |
|  | o__Burkholderiales_unclassified | aerobic | 20 | 1.12 | 0.31 | 0.496 |
|  |  | anaerobic | 20 | 0.82 | 0.31 |  |
|  | f__Enterobacteriaceae_unclassified | aerobic | 20 | 1.91 | 0.61 | 0.521 |
|  |  | anaerobic | 20 | 1.43 | 0.43 |  |
| **Species level** | g__Bifidobacterium_unclassified | aerobic | 20 | 0.94 | 0.48 | 0.742 |
|  |  | anaerobic | 20 | 1.19 | 0.61 |  |
|  | s__aerofaciens | aerobic | 20 | 2.38 | 1.02 | 0.863 |
|  |  | anaerobic | 20 | 2.18 | 0.52 |  |
|  | g__Bacteroides_unclassified | aerobic | 20 | 8.92 | 2.01 | 0.740 |
|  |  | anaerobic | 20 | 7.98 | 1.98 |  |
|  | s__plebeius | aerobic | 20 | 1.23 | 0.66 | 0.392 |
|  |  | anaerobic | 20 | 0.63 | 0.21 |  |
|  | s__copri | aerobic | 20 | 5.89 | 2.33 | 0.598 |
|  |  | anaerobic | 20 | 4.30 | 1.89 |  |
|  | s__stercorea | aerobic | 20 | 1.77 | 1.01 | 0.877 |
|  |  | anaerobic | 20 | 2.00 | 1.08 |  |
|  | s__putredinis | aerobic | 20 | 1.17 | 0.33 | 0.985 |
|  |  | anaerobic | 20 | 1.18 | 0.35 |  |
|  | f__Lachnospiraceae_unclassified | aerobic | 20 | 4.30 | 0.57 | 0.178 |
|  |  | anaerobic | 20 | 5.44 | 0.60 |  |
|  | g__Blautia_unclassified | aerobic | 20 | 2.32 | 0.70 | 0.511 |
|  |  | anaerobic | 20 | 2.96 | 0.65 |  |
|  | g__Coprococcus_unclassified | aerobic | 20 | 1.38 | 0.26 | 0.199 |
|  |  | anaerobic | 20 | 2.05 | 0.45 |  |
|  | g__Lachnospira_unclassified | aerobic | 20 | 1.96 | 0.48 | 0.537 |
|  |  | anaerobic | 20 | 2.69 | 1.06 |  |
|  | g__Roseburia_unclassified | aerobic | 20 | 1.80 | 0.47 | 0.351 |
|  |  | anaerobic | 20 | 2.66 | 0.79 |  |
|  | s__inulinivorans | aerobic | 20 | 1.43 | 0.43 | 0.886 |
|  |  | anaerobic | 20 | 1.52 | 0.45 |  |
|  | f__Ruminococcaceae_unclassified | aerobic | 20 | 3.28 | 0.74 | 0.985 |
|  |  | anaerobic | 20 | 3.30 | 0.81 |  |
|  | s__prausnitzii | aerobic | 20 | 11.35 | 1.40 | 0.546 |
|  |  | anaerobic | 20 | 12.58 | 1.45 |  |
|  | g__Oscillospira_unclassified | aerobic | 20 | 4.18 | 0.92 | 0.963 |
|  |  | anaerobic | 20 | 4.24 | 0.93 |  |
|  | g__Ruminococcus_unclassified | aerobic | 20 | 2.04 | 0.43 | 0.732 |
|  |  | anaerobic | 20 | 1.82 | 0.45 |  |
|  | g__Megamonas_unclassified | aerobic | 20 | 3.34 | 1.79 | 0.697 |
|  |  | anaerobic | 20 | 4.64 | 2.81 |  |
|  | g__Phascolarctobacterium_unclassified | aerobic | 20 | 1.98 | 1.00 | 0.482 |
|  |  | anaerobic | 20 | 1.22 | 0.41 |  |
|  | o__Clostridiales_unclassified | aerobic | 20 | 1.40 | 0.35 | 0.962 |
|  |  | anaerobic | 20 | 1.42 | 0.40 |  |
|  | g__Fusobacterium_unclassified | aerobic | 20 | 2.33 | 1.17 | 0.762 |
|  |  | anaerobic | 20 | 1.88 | 0.92 |  |
|  | g__Sutterella_unclassified | aerobic | 20 | 9.38 | 2.14 | 0.313 |
|  |  | anaerobic | 20 | 6.48 | 1.87 |  |
|  | o__Burkholderiales_unclassified | aerobic | 20 | 1.12 | 0.31 | 0.496 |
|  |  | anaerobic | 20 | 0.82 | 0.31 |  |
|  | f__Enterobacteriaceae_unclassified | aerobic | 20 | 1.91 | 0.61 | 0.521 |
|  |  | anaerobic | 20 | 1.43 | 0.43 |  |
