# Supplementary material for: Non-significant influence between aerobic and anaerobic sample transport materials on gut (fecal) microbiota in healthy and fat-metabolic disorder Thai adults
Source: PeerJ. 2024 Apr 19;12:e17270. doi: 10.7717/peerj.17270 (PMC11034497; doi:10.7717/peerj.17270)
Supplement: Supplemental Information 7 [file peerj-12-17270-s007.docx]

**Table S3.** HOMOVA and AMOVA tests to determine quantitative microbiota community differences at genus and species OTUs between aerobic and anaerobic sample transport groups based on differing beta diversity coefficients (no statistical difference was reported, P > 0.05).

| **Beta diversity coefficients** | **Genus** | | **Species** | |
| --- | --- | --- | --- | --- |
|  | **HOMOVA** | **AMOVA** | **HOMOVA** | **AMOVA** |
| **Thetan** | **1.000** | **0.681** | **1.000** | **0.864** |
| **Sorabund** | **1.000** | **0.604** | **1.000** | **0.809** |
| **Morisita–Horn** | **1.000** | **0.870** | **1.000** | **0.863** |
| **Thetayc** | **1.000** | **0.979** | **1.000** | **0.977** |
| **Bray-Curtis** | **1.000** | **0.221** | **1.000** | **0.308** |
| **Jclass** | **1.000** | **0.980** | **1.000** | **1.000** |
| **Lennon** | **1.000** | **0.716** | **1.000** | **0.801** |
