# Supplementary material for: Non-significant influence between aerobic and anaerobic sample transport materials on gut (fecal) microbiota in healthy and fat-metabolic disorder Thai adults
Source: PeerJ. 2024 Apr 19;12:e17270. doi: 10.7717/peerj.17270 (PMC11034497; doi:10.7717/peerj.17270)
Supplement: Supplemental Information 8 [file peerj-12-17270-s008.docx]

**Bioinformatic and statistical analyses for bacterial microbiota diversity and potential metabolisms**

**Processing of raw sequences to quality sequences**

set.current(processors=8)

#make.file(inputdir=., type=fastq, prefix=stability)

#make.contigs(file=stability.files, processors=8)

#summary.seqs(fasta=stability.trim.contigs.fasta, processors=8)

#screen.seqs(fasta=stability.trim.contigs.fasta,group=stability.contigs.groups, summary=stability.trim.contigs.summary, maxambig=8, minlength=100, processors=8)

#summary.seqs(fasta=stability.trim.contigs.good.fasta)

#unique.seqs(fasta=stability.trim.contigs.good.fasta)

#count.seqs(name=stability.trim.contigs.good.names, group=stability.contigs.good.groups)

#summary.seqs(count=stability.trim.contigs.good.count_table)

#align.seqs(fasta=stability.trim.contigs.good.unique.fasta, reference=silva.nr_v132.align, flip=T)

#summary.seqs(fasta=stability.trim.contigs.good.unique.align, count=stability.trim.contigs.good.count_table)

#screen.seqs(fasta=stability.trim.contigs.good.unique.align,count=stability.trim.contigs.good.count_table,summary=stability.trim.contigs.good.unique.summary,start=6450,end=26994,maxhomop=8,processors=8)

#summary.seqs(fasta=stability.trim.contigs.good.unique.good.align, count=stability.trim.contigs.good.good.count_table)

#filter.seqs(fasta=stability.trim.contigs.good.unique.good.align,vertical=T,trump=.,processors=8)

#unique.seqs(fasta=stability.trim.contigs.good.unique.good.filter.fasta, count=stability.trim.contigs.good.good.count_table)

#pre.cluster(fasta=stability.trim.contigs.good.unique.good.filter.unique.fasta, count=stability.trim.contigs.good.unique.good.filter.count_table, diffs=2)

#chimera.vsearch(fasta=stability.trim.contigs.good.unique.good.filter.unique.precluster.fasta, count=stability.trim.contigs.good.unique.good.filter.unique.precluster.count_table, dereplicate=t)

#remove.seqs(fasta=stability.trim.contigs.good.unique.good.filter.unique.precluster.fasta, accnos=stability.trim.contigs.good.unique.good.filter.unique.precluster.denovo.vsearch.accnos)

#summary.seqs(fasta=current, count=current)

#classify.seqs(fasta=stability.trim.contigs.good.unique.good.filter.unique.precluster.pick.fasta, count=stability.trim.contigs.good.unique.good.filter.unique.precluster.denovo.vsearch.pick.count_table, reference=gg_13_8_99.fasta, taxonomy=gg_13_8_99.gg.tax, cutoff=80)

#remove.lineage(fasta=stability.trim.contigs.good.unique.good.filter.unique.precluster.pick.fasta, count=stability.trim.contigs.good.unique.good.filter.unique.precluster.denovo.vsearch.pick.count_table, taxonomy=stability.trim.contigs.good.unique.good.filter.unique.precluster.pick.gg.wang.taxonomy, taxon=Chloroplast-Mitochondria-unknown-Archaea-Eukaryota)

#summary.tax(taxonomy=stability.trim.contigs.good.unique.good.filter.unique.precluster.pick.gg.wang.pick.taxonomy, count=stability.trim.contigs.good.unique.good.filter.unique.precluster.denovo.vsearch.pick.pick.count_table)

#system(cp stability.trim.contigs.good.unique.good.filter.unique.precluster.pick.pick.fasta 20.fasta)

#system(cp stability.trim.contigs.good.unique.good.filter.unique.precluster.denovo.vsearch.pick.pick.count_table 20.count_table)

#system(cp stability.trim.contigs.good.unique.good.filter.unique.precluster.pick.gg.wang.pick.taxonomy 20.taxonomy)

#make.shared(list=20.fn.unique_list.list, count=20.count_table, label=0.03)

**Phylotype classification to OTU and sample sequence normalization**

#phylotype(taxonomy=20.taxonomy)

#make.shared(list=20.tx.list, count=20.count_table, label=1-2-3-4-5-6)

#classify.otu(list=20.tx.list, count=20.count_table,taxonomy=20.taxonomy, basis=sequence, label=1-2-3-4-5-6)

#recheck

#classify.otu(list=20.tx.list, count=20.count_table,taxonomy=20.taxonomy, basis=sequence, label=1)

#count.groups(shared=20.tx.shared)

#sub.sample(shared=20.tx.shared, size=7137)

#collect.single(shared=20.tx.shared, calc=chao, freq=100)

**Rarefaction curve and alpha diversity analysis**

#rarefaction.single(shared=20.tx.1.subsample.shared, calc=sobs, freq=100,processors=8)

#summary.single(shared=20.tx.1.subsample.shared,calc=nseqs-coverage-sobs-invsimpson-chao-shannon-npshannon)

#rarefaction.single(shared=20.tx.2.subsample.shared, calc=sobs, freq=100,processors=8)

#summary.single(shared=20.tx.2.subsample.shared,calc=nseqs-coverage-sobs-invsimpson-chao-shannon-npshannon)

#rarefaction.single(shared=20.tx.6.subsample.shared, calc=sobs, freq=100,processors=8)

#summary.single(shared=20.tx.6.subsample.shared,calc=nseqs-coverage-sobs-invsimpson-chao-shannon-npshannon)

#rarefaction.single(shared=20.tx.5.subsample.shared, calc=sobs, freq=100,processors=8)

#summary.single(shared=20.tx.5.subsample.shared,calc=nseqs-coverage-sobs-invsimpson-chao-shannon-npshannon)

#rarefaction.single(shared=20.tx.5.subsample.shared, calc=sobs, freq=100,processors=8)

#summary.single(shared=20.tx.5.subsample.shared,calc=nseqs-coverage-sobs-invsimpson-chao-shannon-npshannon)

#dist.shared(shared=final.tx.1.subsample.shared,calc=lennon-jclass-morisitahorn-sorabund-thetan-thetayc-braycurtis)

#summary.shared(shared=final.tx.1.subsample.shared, calc=braycurtis)

**Beta diversity analysis using different dissimilarity metrics**

dist.shared(shared=edith20.tx.1.subsample.shared, calc=lennon-jclass-morisitahorn-sorabund-thetan-thetayc-braycurtis)

dist.shared(shared=edith20.tx.2.subsample.shared, calc=lennon-jclass-morisitahorn-sorabund-thetan-thetayc-braycurtis)

dist.shared(shared=edit20.tx.1.subsample.shared, calc=lennon-jclass-morisitahorn-sorabund-thetan-thetayc-braycurtis)

dist.shared(shared=edit20.tx.2.subsample.shared, calc=lennon-jclass-morisitahorn-sorabund-thetan-thetayc-braycurtis)

dist.shared(shared=newcopies20.tx.1.subsample.shared, calc=lennon-jclass-morisitahorn-sorabund-thetan-thetayc-braycurtis)

dist.shared(shared=newcopies20.tx.2.subsample.shared, calc=lennon-jclass-morisitahorn-sorabund-thetan-thetayc-braycurtis)

dist.shared(shared=hnewcopies20.tx.2.subsample.shared, calc=lennon-jclass-morisitahorn-sorabund-thetan-thetayc-braycurtis)

dist.shared(shared=hnewcopies20.tx.1.subsample.shared, calc=lennon-jclass-morisitahorn-sorabund-thetan-thetayc-braycurtis)

nmds(phylip=hnewcopies20.tx.1.subsample.braycurtis.1.lt.dist, mindim=2, maxdim=2)

nmds(phylip=hnewcopies20.tx.1.subsample.jclass.1.lt.dist, mindim=2, maxdim=2)

nmds(phylip=newcopies20.tx.1.subsample.lennon.1.lt.dist, mindim=2, maxdim=2)

nmds(phylip=hnewcopies20.tx.1.subsample.morisitahorn.1.lt.dist, mindim=2, maxdim=2)

nmds(phylip=hnewcopies20.tx.1.subsample.sorabund.1.lt.dist, mindim=2, maxdim=2)

nmds(phylip=hnewcopies20.tx.1.subsample.thetan.1.lt.dist, mindim=2, maxdim=2)

nmds(phylip=hnewcopies20.tx.1.subsample.thetayc.1.lt.dist, mindim=2, maxdim=2)

nmds(phylip=edith20.tx.1.subsample.braycurtis.1.lt.dist, mindim=2, maxdim=2)

nmds(phylip=edith20.tx.1.subsample.jclass.1.lt.dist, mindim=2, maxdim=2)

nmds(phylip=edith20.tx.1.subsample.lennon.1.lt.dist, mindim=2, maxdim=2)

nmds(phylip=edith20.tx.1.subsample.morisitahorn.1.lt.dist, mindim=2, maxdim=2)

nmds(phylip=edith20.tx.1.subsample.sorabund.1.lt.dist, mindim=2, maxdim=2)

nmds(phylip=edith20.tx.1.subsample.thetan.1.lt.dist, mindim=2, maxdim=2)

nmds(phylip=edith20.tx.1.subsample.thetayc.1.lt.dist, mindim=2, maxdim=2)

corr.axes(axes=hnewcopies20.tx.1.subsample.thetan.1.lt.nmds.axes, shared=hnewcopies20.tx.1.subsample, method=pearson, numaxes=2, label=1)

corr.axes(axes=newcopies20.tx.1.subsample.thetan.1.lt.nmds.axes, metadata=gut20health.metadata, method=pearson, numaxes=2, label=1)

**LEfSe analysis and LDA differences between subject groups**

lefse(shared=hnewcopies20.tx.1.subsample.shared, design=20healthlefse.design, constaxonomy=20.tx.1.cons.taxonomy)

lefse(shared=hnewcopies20.tx.1.subsample.shared, design=20healthlefse.design)

lefse(shared=metabolic.txt, design=metaboliclefse.design)

**Metabolic potential predictions**

#make.biom(shared=healthhnewcopies20.tx.1.subsample.shared, label=1, reftaxonomy=gg_13_8_99.gg.tax, constaxonomy=20.tx.1.cons.taxonomy, picrust=97_otu_map.txt)

##Activate picrust

source /colossus/home/suchittra/miniconda2/bin/activate picrust1

export PATH=/colossus/home/suchittra/miniconda2/bin:/colossus/home/suchittra/miniconda2/envs/picrust1/bin:$PATH

##Functional potential prediction

biom convert --table-type="OTU table" -i rawhnewcopies20.tx.1.subsample.1.biom -o rawhnewcopies20.tx.1.subsample.1.txt --to-tsv --header-key taxonomy

biom convert -i rawhnewcopies20.tx.1.subsample.1.txt -o rawhnewcopies20.tx.1.subsample.1.1json.biom --table-type="OTU table" --to-json --process-obs-metadata taxonomy

normalize_by_copy_number.py -i rawhnewcopies20.tx.1.subsample.1.1json.biom -o rawhnewcopies20.tx.1.subsample.1.biom

predict_metagenomes.py -i rawhnewcopies20.tx.1.subsample.1.biom -o rawhnewcopies20.tx.1.subsample.1.biom

categorize_by_function.py -i rawhnewcopies20.tx.1.subsample.1.biom -c KEGG_Pathways -l 2 -o rawhnewcopies20.tx.1.subsample.1.L1.biom

biom convert -i rawhnewcopies20.tx.1.subsample.1.L1.biom -o rawhnewcopies20.tx.1.subsample.1.L2json.biom --to-json

biom convert --table-type="OTU table" -i Unotu33hnewcopies20.tx.1.subsample.1.biom -o Unotu33hnewcopies20.tx.1.subsample.1.txt --to-tsv --header-key taxonomy

biom convert -i Unotu33hnewcopies20.tx.1.subsample.1.txt -o Unotu33hnewcopies20.tx.1.subsample.1.1json.biom --table-type="OTU table" --to-json --process-obs-metadata taxonomy

normalize_by_copy_number.py -i Unotu33hnewcopies20.tx.1.subsample.1.1json.biom -o Unotu33hnewcopies20.tx.1.subsample.1.biom

predict_metagenomes.py -i Unotu33hnewcopies20.tx.1.subsample.1.biom -o Unotu33hnewcopies20.tx.1.subsample.1.biom

categorize_by_function.py -i All33hnewcopies20.tx.1.subsample.1.biom -c KEGG_Pathways -l 2 -o All33hnewcopies20.tx.1.subsample.1.L1.biom

biom convert -i All33hnewcopies20.tx.1.subsample.1.L1.biom -o All33hnewcopies20.tx.1.subsample.1.L2json.biom --to-json

biom convert --table-type="OTU table" -i All8hnewcopies20.tx.1.subsample.1.biom -o All8hnewcopies20.tx.1.subsample.1.txt --to-tsv --header-key taxonomy

biom convert -i All8hnewcopies20.tx.1.subsample.1.txt -o All8hnewcopies20.tx.1.subsample.1.1json.biom --table-type="OTU table" --to-json --process-obs-metadata taxonomy

normalize_by_copy_number.py -i All8hnewcopies20.tx.1.subsample.1.1json.biom -o All8hnewcopies20.tx.1.subsample.1.biom

predict_metagenomes.py -i All8hnewcopies20.tx.1.subsample.1.biom -o All8hnewcopies20.tx.1.subsample.1.biom

categorize_by_function.py -i All8hnewcopies20.tx.1.subsample.1.biom -c KEGG_Pathways -l 2 -o All8hnewcopies20.tx.1.subsample.1.L1.biom

biom convert -i All8hnewcopies20.tx.1.subsample.1.L1.biom -o All8hnewcopies20.tx.1.subsample.1.L2json.biom --to-json

biom convert --table-type="OTU table" -i All123hnewcopies20.tx.1.subsample.1.biom -o All123hnewcopies20.tx.1.subsample.1.txt --to-tsv --header-key taxonomy

biom convert -i All123hnewcopies20.tx.1.subsample.1.txt -o All123hnewcopies20.tx.1.subsample.1.1json.biom --table-type="OTU table" --to-json --process-obs-metadata taxonomy

normalize_by_copy_number.py -i All123hnewcopies20.tx.1.subsample.1.1json.biom -o All123hnewcopies20.tx.1.subsample.1.biom

predict_metagenomes.py -i All123hnewcopies20.tx.1.subsample.1.biom -o All123hnewcopies20.tx.1.subsample.1.biom

categorize_by_function.py -i All123hnewcopies20.tx.1.subsample.1.biom -c KEGG_Pathways -l 2 -o All123hnewcopies20.tx.1.subsample.1.L1.biom

biom convert -i All123hnewcopies20.tx.1.subsample.1.L1.biom -o All123hnewcopies20.tx.1.subsample.1.L2json.biom --to-json

biom convert --table-type="OTU table" -i All24hnewcopies20.tx.1.subsample.1.biom -o All24hnewcopies20.tx.1.subsample.1.txt --to-tsv --header-key taxonomy

biom convert -i All24hnewcopies20.tx.1.subsample.1.txt -o All24hnewcopies20.tx.1.subsample.1.1json.biom --table-type="OTU table" --to-json --process-obs-metadata taxonomy

normalize_by_copy_number.py -i All24hnewcopies20.tx.1.subsample.1.1json.biom -o All24hnewcopies20.tx.1.subsample.1.biom

predict_metagenomes.py -i All24hnewcopies20.tx.1.subsample.1.biom -o All24hnewcopies20.tx.1.subsample.1.biom

categorize_by_function.py -i All24hnewcopies20.tx.1.subsample.1.biom -c KEGG_Pathways -l 2 -o All24hnewcopies20.tx.1.subsample.1.L1.biom

biom convert -i All24hnewcopies20.tx.1.subsample.1.L1.biom -o All24hnewcopies20.tx.1.subsample.1.L2json.biom --to-json

biom convert --table-type="OTU table" -i All103hnewcopies20.tx.1.subsample.1.biom -o All103hnewcopies20.tx.1.subsample.1.txt --to-tsv --header-key taxonomy

biom convert -i All103hnewcopies20.tx.1.subsample.1.txt -o All103hnewcopies20.tx.1.subsample.1.1json.biom --table-type="OTU table" --to-json --process-obs-metadata taxonomy

normalize_by_copy_number.py -i All103hnewcopies20.tx.1.subsample.1.1json.biom -o All103hnewcopies20.tx.1.subsample.1.biom

predict_metagenomes.py -i All103hnewcopies20.tx.1.subsample.1.biom -o All103hnewcopies20.tx.1.subsample.1.biom

categorize_by_function.py -i All103hnewcopies20.tx.1.subsample.1.biom -c KEGG_Pathways -l 2 -o All103hnewcopies20.tx.1.subsample.1.L1.biom

biom convert -i All103hnewcopies20.tx.1.subsample.1.L1.biom -o All103hnewcopies20.tx.1.subsample.1.L2json.biom --to-json

biom convert --table-type="OTU table" -i All175hnewcopies20.tx.1.subsample.1.biom -o All175hnewcopies20.tx.1.subsample.1.txt --to-tsv --header-key taxonomy

biom convert -i All175hnewcopies20.tx.1.subsample.1.txt -o All175hnewcopies20.tx.1.subsample.1.1json.biom --table-type="OTU table" --to-json --process-obs-metadata taxonomy

normalize_by_copy_number.py -i All175hnewcopies20.tx.1.subsample.1.1json.biom -o All175hnewcopies20.tx.1.subsample.1.biom

predict_metagenomes.py -i All175hnewcopies20.tx.1.subsample.1.biom -o All175hnewcopies20.tx.1.subsample.1.biom

categorize_by_function.py -i All175hnewcopies20.tx.1.subsample.1.biom -c KEGG_Pathways -l 2 -o All175hnewcopies20.tx.1.subsample.1.L1.biom

biom convert -i All175hnewcopies20.tx.1.subsample.1.L1.biom -o All175hnewcopies20.tx.1.subsample.1.L2json.biom --to-json

biom convert --table-type="OTU table" -i All30hnewcopies20.tx.1.subsample.1.biom -o All30hnewcopies20.tx.1.subsample.1.txt --to-tsv --header-key taxonomy

biom convert -i All30hnewcopies20.tx.1.subsample.1.txt -o All30hnewcopies20.tx.1.subsample.1.1json.biom --table-type="OTU table" --to-json --process-obs-metadata taxonomy

normalize_by_copy_number.py -i All30hnewcopies20.tx.1.subsample.1.1json.biom -o All30hnewcopies20.tx.1.subsample.1.biom

predict_metagenomes.py -i All30hnewcopies20.tx.1.subsample.1.biom -o All30hnewcopies20.tx.1.subsample.1.biom

categorize_by_function.py -i All30hnewcopies20.tx.1.subsample.1.biom -c KEGG_Pathways -l 2 -o All30hnewcopies20.tx.1.subsample.1.L1.biom

biom convert -i All30hnewcopies20.tx.1.subsample.1.L1.biom -o All30hnewcopies20.tx.1.subsample.1.L2json.biom --to-json

biom convert --table-type="OTU table" -i All88hnewcopies20.tx.1.subsample.1.biom -o All88hnewcopies20.tx.1.subsample.1.txt --to-tsv --header-key taxonomy

biom convert -i All88hnewcopies20.tx.1.subsample.1.txt -o All88hnewcopies20.tx.1.subsample.1.1json.biom --table-type="OTU table" --to-json --process-obs-metadata taxonomy

normalize_by_copy_number.py -i All88hnewcopies20.tx.1.subsample.1.1json.biom -o All88hnewcopies20.tx.1.subsample.1.biom

predict_metagenomes.py -i All88hnewcopies20.tx.1.subsample.1.biom -o All88hnewcopies20.tx.1.subsample.1.biom

categorize_by_function.py -i All88hnewcopies20.tx.1.subsample.1.biom -c KEGG_Pathways -l 2 -o All88hnewcopies20.tx.1.subsample.1.L1.biom

biom convert -i All88hnewcopies20.tx.1.subsample.1.L1.biom -o All88hnewcopies20.tx.1.subsample.1.L2json.biom --to-json

biom convert --table-type="OTU table" -i All75hnewcopies20.tx.1.subsample.1.biom -o All75hnewcopies20.tx.1.subsample.1.txt --to-tsv --header-key taxonomy

biom convert -i All75hnewcopies20.tx.1.subsample.1.txt -o All75hnewcopies20.tx.1.subsample.1.1json.biom --table-type="OTU table" --to-json --process-obs-metadata taxonomy

normalize_by_copy_number.py -i All75hnewcopies20.tx.1.subsample.1.1json.biom -o All75hnewcopies20.tx.1.subsample.1.biom

predict_metagenomes.py -i All75hnewcopies20.tx.1.subsample.1.biom -o All75hnewcopies20.tx.1.subsample.1.biom

categorize_by_function.py -i All75hnewcopies20.tx.1.subsample.1.biom -c KEGG_Pathways -l 2 -o All75hnewcopies20.tx.1.subsample.1.L1.biom

biom convert -i All75hnewcopies20.tx.1.subsample.1.L1.biom -o All75hnewcopies20.tx.1.subsample.1.L2json.biom --to-json

biom convert --table-type="OTU table" -i All27hnewcopies20.tx.1.subsample.1.biom -o All27hnewcopies20.tx.1.subsample.1.txt --to-tsv --header-key taxonomy

biom convert -i All27hnewcopies20.tx.1.subsample.1.txt -o All27hnewcopies20.tx.1.subsample.1.1json.biom --table-type="OTU table" --to-json --process-obs-metadata taxonomy

normalize_by_copy_number.py -i All27hnewcopies20.tx.1.subsample.1.1json.biom -o All27hnewcopies20.tx.1.subsample.1.biom

predict_metagenomes.py -i All27hnewcopies20.tx.1.subsample.1.biom -o All27hnewcopies20.tx.1.subsample.1.biom

categorize_by_function.py -i All27hnewcopies20.tx.1.subsample.1.biom -c KEGG_Pathways -l 2 -o All27hnewcopies20.tx.1.subsample.1.L1.biom

biom convert -i All27hnewcopies20.tx.1.subsample.1.L1.biom -o All27hnewcopies20.tx.1.subsample.1.L2json.biom --to-json

biom convert --table-type="OTU table" -i All4hnewcopies20.tx.1.subsample.1.biom -o All4hnewcopies20.tx.1.subsample.1.txt --to-tsv --header-key taxonomy

biom convert -i All4hnewcopies20.tx.1.subsample.1.txt -o All4hnewcopies20.tx.1.subsample.1.1json.biom --table-type="OTU table" --to-json --process-obs-metadata taxonomy

normalize_by_copy_number.py -i All4hnewcopies20.tx.1.subsample.1.1json.biom -o All4hnewcopies20.tx.1.subsample.1.biom

predict_metagenomes.py -i All4hnewcopies20.tx.1.subsample.1.biom -o All4hnewcopies20.tx.1.subsample.1.biom

categorize_by_function.py -i All4hnewcopies20.tx.1.subsample.1.biom -c KEGG_Pathways -l 2 -o All4hnewcopies20.tx.1.subsample.1.L1.biom

biom convert -i All4hnewcopies20.tx.1.subsample.1.L1.biom -o All4hnewcopies20.tx.1.subsample.1.L2json.biom --to-json

biom convert --table-type="OTU table" -i All13hnewcopies20.tx.1.subsample.1.biom -o All13hnewcopies20.tx.1.subsample.1.txt --to-tsv --header-key taxonomy

biom convert -i All13hnewcopies20.tx.1.subsample.1.txt -o All13hnewcopies20.tx.1.subsample.1.1json.biom --table-type="OTU table" --to-json --process-obs-metadata taxonomy

normalize_by_copy_number.py -i All13hnewcopies20.tx.1.subsample.1.1json.biom -o All13hnewcopies20.tx.1.subsample.1.biom

predict_metagenomes.py -i All13hnewcopies20.tx.1.subsample.1.biom -o All13hnewcopies20.tx.1.subsample.1.biom

categorize_by_function.py -i All13hnewcopies20.tx.1.subsample.1.biom -c KEGG_Pathways -l 2 -o All13hnewcopies20.tx.1.subsample.1.L1.biom

biom convert -i All13hnewcopies20.tx.1.subsample.1.L1.biom -o All13hnewcopies20.tx.1.subsample.1.L2json.biom --to-json

biom convert --table-type="OTU table" -i All111hnewcopies20.tx.1.subsample.1.biom -o All111hnewcopies20.tx.1.subsample.1.txt --to-tsv --header-key taxonomy

biom convert -i All111hnewcopies20.tx.1.subsample.1.txt -o All111hnewcopies20.tx.1.subsample.1.1json.biom --table-type="OTU table" --to-json --process-obs-metadata taxonomy

normalize_by_copy_number.py -i All111hnewcopies20.tx.1.subsample.1.1json.biom -o All111hnewcopies20.tx.1.subsample.1.biom

predict_metagenomes.py -i All111hnewcopies20.tx.1.subsample.1.biom -o All111hnewcopies20.tx.1.subsample.1.biom

categorize_by_function.py -i All111hnewcopies20.tx.1.subsample.1.biom -c KEGG_Pathways -l 2 -o All111hnewcopies20.tx.1.subsample.1.L1.biom

biom convert -i All111hnewcopies20.tx.1.subsample.1.L1.biom -o All111hnewcopies20.tx.1.subsample.1.L2json.biom --to-json

biom convert --table-type="OTU table" -i All3hnewcopies20.tx.1.subsample.1.biom -o All3hnewcopies20.tx.1.subsample.1.txt --to-tsv --header-key taxonomy

biom convert -i All3hnewcopies20.tx.1.subsample.1.txt -o All3hnewcopies20.tx.1.subsample.1.1json.biom --table-type="OTU table" --to-json --process-obs-metadata taxonomy

normalize_by_copy_number.py -i All3hnewcopies20.tx.1.subsample.1.1json.biom -o All3hnewcopies20.tx.1.subsample.1.biom

predict_metagenomes.py -i All33hnewcopies20.tx.1.subsample.1.biom -o All3hnewcopies20.tx.1.subsample.1.biom

categorize_by_function.py -i All3hnewcopies20.tx.1.subsample.1.biom -c KEGG_Pathways -l 2 -o All3hnewcopies20.tx.1.subsample.1.L1.biom

biom convert -i All3hnewcopies20.tx.1.subsample.1.L1.biom -o All3hnewcopies20.tx.1.subsample.1.L2json.biom --to-json

biom convert --table-type="OTU table" -i All47hnewcopies20.tx.1.subsample.1.biom -o All47hnewcopies20.tx.1.subsample.1.txt --to-tsv --header-key taxonomy

biom convert -i All47hnewcopies20.tx.1.subsample.1.txt -o All47hnewcopies20.tx.1.subsample.1.1json.biom --table-type="OTU table" --to-json --process-obs-metadata taxonomy

normalize_by_copy_number.py -i All47hnewcopies20.tx.1.subsample.1.1json.biom -o All47hnewcopies20.tx.1.subsample.1.biom

predict_metagenomes.py -i All47hnewcopies20.tx.1.subsample.1.biom -o All47hnewcopies20.tx.1.subsample.1.biom

categorize_by_function.py -i All47hnewcopies20.tx.1.subsample.1.biom -c KEGG_Pathways -l 2 -o All47hnewcopies20.tx.1.subsample.1.L1.biom

biom convert -i All47hnewcopies20.tx.1.subsample.1.L1.biom -o All47hnewcopies20.tx.1.subsample.1.L2json.biom --to-json

biom convert --table-type="OTU table" -i Unotu33hnewcopies20.tx.1.subsample.1.biom -o Unotu33hnewcopies20.tx.1.subsample.1.txt --to-tsv --header-key taxonomy

biom convert -i Unotu33hnewcopies20.tx.1.subsample.1.txt -o Unotu33hnewcopies20.tx.1.subsample.1.1json.biom --table-type="OTU table" --to-json --process-obs-metadata taxonomy

normalize_by_copy_number.py -i Unotu33hnewcopies20.tx.1.subsample.1.1json.biom -o Unotu33hnewcopies20.tx.1.subsample.1.biom

predict_metagenomes.py -i Unotu33hnewcopies20.tx.1.subsample.1.biom -o Unotu33hnewcopies20.tx.1.subsample.1.biom

categorize_by_function.py -i Unotu33hnewcopies20.tx.1.subsample.1.biom -c KEGG_Pathways -l 2 -o Unotu33hnewcopies20.tx.1.subsample.1.L1.biom

biom convert -i Unotu33hnewcopies20.tx.1.subsample.1.L1.biom -o Unotu33hnewcopies20.tx.1.subsample.1.L2json.biom --to-json

biom convert --table-type="OTU table" -i Unotu8hnewcopies20.tx.1.subsample.1.biom -o Unotu8hnewcopies20.tx.1.subsample.1.txt --to-tsv --header-key taxonomy

biom convert -i Unotu8hnewcopies20.tx.1.subsample.1.txt -o Unotu8hnewcopies20.tx.1.subsample.1.1json.biom --table-type="OTU table" --to-json --process-obs-metadata taxonomy

normalize_by_copy_number.py -i Unotu8hnewcopies20.tx.1.subsample.1.1json.biom -o Unotu8hnewcopies20.tx.1.subsample.1.biom

predict_metagenomes.py -i Unotu8hnewcopies20.tx.1.subsample.1.biom -o Unotu8hnewcopies20.tx.1.subsample.1.biom

categorize_by_function.py -i Unotu8hnewcopies20.tx.1.subsample.1.biom -c KEGG_Pathways -l 2 -o Unotu8hnewcopies20.tx.1.subsample.1.L1.biom

biom convert -i Unotu8hnewcopies20.tx.1.subsample.1.L1.biom -o Unotu8hnewcopies20.tx.1.subsample.1.L2json.biom --to-json

biom convert --table-type="OTU table" -i Unotu123hnewcopies20.tx.1.subsample.1.biom -o Unotu123hnewcopies20.tx.1.subsample.1.txt --to-tsv --header-key taxonomy

biom convert -i Unotu123hnewcopies20.tx.1.subsample.1.txt -o Unotu123hnewcopies20.tx.1.subsample.1.1json.biom --table-type="OTU table" --to-json --process-obs-metadata taxonomy

normalize_by_copy_number.py -i Unotu123hnewcopies20.tx.1.subsample.1.1json.biom -o Unotu123hnewcopies20.tx.1.subsample.1.biom

predict_metagenomes.py -i Unotu123hnewcopies20.tx.1.subsample.1.biom -o Unotu123hnewcopies20.tx.1.subsample.1.biom

categorize_by_function.py -i Unotu123hnewcopies20.tx.1.subsample.1.biom -c KEGG_Pathways -l 2 -o Unotu123hnewcopies20.tx.1.subsample.1.L1.biom

biom convert -i Unotu123hnewcopies20.tx.1.subsample.1.L1.biom -o Unotu123hnewcopies20.tx.1.subsample.1.L2json.biom --to-json

biom convert --table-type="OTU table" -i Unotu24hnewcopies20.tx.1.subsample.1.biom -o Unotu24hnewcopies20.tx.1.subsample.1.txt --to-tsv --header-key taxonomy

biom convert -i Unotu24hnewcopies20.tx.1.subsample.1.txt -o Unotu24hnewcopies20.tx.1.subsample.1.1json.biom --table-type="OTU table" --to-json --process-obs-metadata taxonomy

normalize_by_copy_number.py -i Unotu24hnewcopies20.tx.1.subsample.1.1json.biom -o Unotu24hnewcopies20.tx.1.subsample.1.biom

predict_metagenomes.py -i Unotu24hnewcopies20.tx.1.subsample.1.biom -o Unotu24hnewcopies20.tx.1.subsample.1.biom

categorize_by_function.py -i Unotu24hnewcopies20.tx.1.subsample.1.biom -c KEGG_Pathways -l 2 -o Unotu24hnewcopies20.tx.1.subsample.1.L1.biom

biom convert -i Unotu24hnewcopies20.tx.1.subsample.1.L1.biom -o Unotu24hnewcopies20.tx.1.subsample.1.L2json.biom --to-json

biom convert --table-type="OTU table" -i Unotu103hnewcopies20.tx.1.subsample.1.biom -o Unotu103hnewcopies20.tx.1.subsample.1.txt --to-tsv --header-key taxonomy

biom convert -i Unotu103hnewcopies20.tx.1.subsample.1.txt -o Unotu103hnewcopies20.tx.1.subsample.1.1json.biom --table-type="OTU table" --to-json --process-obs-metadata taxonomy

normalize_by_copy_number.py -i Unotu103hnewcopies20.tx.1.subsample.1.1json.biom -o Unotu103hnewcopies20.tx.1.subsample.1.biom

predict_metagenomes.py -i Unotu103hnewcopies20.tx.1.subsample.1.biom -o Unotu103hnewcopies20.tx.1.subsample.1.biom

categorize_by_function.py -i Unotu103hnewcopies20.tx.1.subsample.1.biom -c KEGG_Pathways -l 2 -o Unotu103hnewcopies20.tx.1.subsample.1.L1.biom

biom convert -i Unotu103hnewcopies20.tx.1.subsample.1.L1.biom -o Unotu103hnewcopies20.tx.1.subsample.1.L2json.biom --to-json

biom convert --table-type="OTU table" -i Unotu175hnewcopies20.tx.1.subsample.1.biom -o Unotu175hnewcopies20.tx.1.subsample.1.txt --to-tsv --header-key taxonomy

biom convert -i Unotu175hnewcopies20.tx.1.subsample.1.txt -o Unotu175hnewcopies20.tx.1.subsample.1.1json.biom --table-type="OTU table" --to-json --process-obs-metadata taxonomy

normalize_by_copy_number.py -i Unotu175hnewcopies20.tx.1.subsample.1.1json.biom -o Unotu175hnewcopies20.tx.1.subsample.1.biom

predict_metagenomes.py -i Unotu175hnewcopies20.tx.1.subsample.1.biom -o Unotu175hnewcopies20.tx.1.subsample.1.biom

categorize_by_function.py -i Unotu175hnewcopies20.tx.1.subsample.1.biom -c KEGG_Pathways -l 2 -o Unotu175hnewcopies20.tx.1.subsample.1.L1.biom

biom convert -i Unotu175hnewcopies20.tx.1.subsample.1.L1.biom -o Unotu175hnewcopies20.tx.1.subsample.1.L2json.biom --to-json

biom convert --table-type="OTU table" -i Unotu30hnewcopies20.tx.1.subsample.1.biom -o Unotu30hnewcopies20.tx.1.subsample.1.txt --to-tsv --header-key taxonomy

biom convert -i Unotu30hnewcopies20.tx.1.subsample.1.txt -o Unotu30hnewcopies20.tx.1.subsample.1.1json.biom --table-type="OTU table" --to-json --process-obs-metadata taxonomy

normalize_by_copy_number.py -i Unotu30hnewcopies20.tx.1.subsample.1.1json.biom -o Unotu30hnewcopies20.tx.1.subsample.1.biom

predict_metagenomes.py -i Unotu30hnewcopies20.tx.1.subsample.1.biom -o Unotu30hnewcopies20.tx.1.subsample.1.biom

categorize_by_function.py -i Unotu30hnewcopies20.tx.1.subsample.1.biom -c KEGG_Pathways -l 2 -o Unotu30hnewcopies20.tx.1.subsample.1.L1.biom

biom convert -i Unotu30hnewcopies20.tx.1.subsample.1.L1.biom -o Unotu30hnewcopies20.tx.1.subsample.1.L2json.biom --to-json

biom convert --table-type="OTU table" -i Unotu88hnewcopies20.tx.1.subsample.1.biom -o Unotu88hnewcopies20.tx.1.subsample.1.txt --to-tsv --header-key taxonomy

biom convert -i Unotu88hnewcopies20.tx.1.subsample.1.txt -o Unotu88hnewcopies20.tx.1.subsample.1.1json.biom --table-type="OTU table" --to-json --process-obs-metadata taxonomy

normalize_by_copy_number.py -i Unotu88hnewcopies20.tx.1.subsample.1.1json.biom -o Unotu88hnewcopies20.tx.1.subsample.1.biom

predict_metagenomes.py -i Unotu88hnewcopies20.tx.1.subsample.1.biom -o Unotu88hnewcopies20.tx.1.subsample.1.biom

categorize_by_function.py -i Unotu88hnewcopies20.tx.1.subsample.1.biom -c KEGG_Pathways -l 2 -o Unotu88hnewcopies20.tx.1.subsample.1.L1.biom

biom convert -i Unotu88hnewcopies20.tx.1.subsample.1.L1.biom -o Unotu88hnewcopies20.tx.1.subsample.1.L2json.biom --to-json

biom convert --table-type="OTU table" -i Unotu75hnewcopies20.tx.1.subsample.1.biom -o Unotu75hnewcopies20.tx.1.subsample.1.txt --to-tsv --header-key taxonomy

biom convert -i Unotu75hnewcopies20.tx.1.subsample.1.txt -o Unotu75hnewcopies20.tx.1.subsample.1.1json.biom --table-type="OTU table" --to-json --process-obs-metadata taxonomy

normalize_by_copy_number.py -i Unotu75hnewcopies20.tx.1.subsample.1.1json.biom -o Unotu75hnewcopies20.tx.1.subsample.1.biom

predict_metagenomes.py -i Unotu75hnewcopies20.tx.1.subsample.1.biom -o Unotu75hnewcopies20.tx.1.subsample.1.biom

categorize_by_function.py -i Unotu75hnewcopies20.tx.1.subsample.1.biom -c KEGG_Pathways -l 2 -o Unotu75hnewcopies20.tx.1.subsample.1.L1.biom

biom convert -i Unotu75hnewcopies20.tx.1.subsample.1.L1.biom -o Unotu75hnewcopies20.tx.1.subsample.1.L2json.biom --to-json

biom convert --table-type="OTU table" -i Unotu27hnewcopies20.tx.1.subsample.1.biom -o Unotu27hnewcopies20.tx.1.subsample.1.txt --to-tsv --header-key taxonomy

biom convert -i Unotu27hnewcopies20.tx.1.subsample.1.txt -o Unotu27hnewcopies20.tx.1.subsample.1.1json.biom --table-type="OTU table" --to-json --process-obs-metadata taxonomy

normalize_by_copy_number.py -i Unotu27hnewcopies20.tx.1.subsample.1.1json.biom -o Unotu27hnewcopies20.tx.1.subsample.1.biom

predict_metagenomes.py -i Unotu27hnewcopies20.tx.1.subsample.1.biom -o Unotu27hnewcopies20.tx.1.subsample.1.biom

categorize_by_function.py -i Unotu27hnewcopies20.tx.1.subsample.1.biom -c KEGG_Pathways -l 2 -o Unotu27hnewcopies20.tx.1.subsample.1.L1.biom

biom convert -i Unotu27hnewcopies20.tx.1.subsample.1.L1.biom -o Unotu27hnewcopies20.tx.1.subsample.1.L2json.biom --to-json

biom convert --table-type="OTU table" -i Unotu4hnewcopies20.tx.1.subsample.1.biom -o Unotu4hnewcopies20.tx.1.subsample.1.txt --to-tsv --header-key taxonomy

biom convert -i Unotu4hnewcopies20.tx.1.subsample.1.txt -o Unotu4hnewcopies20.tx.1.subsample.1.1json.biom --table-type="OTU table" --to-json --process-obs-metadata taxonomy

normalize_by_copy_number.py -i Unotu4hnewcopies20.tx.1.subsample.1.1json.biom -o Unotu4hnewcopies20.tx.1.subsample.1.biom

predict_metagenomes.py -i Unotu4hnewcopies20.tx.1.subsample.1.biom -o Unotu4hnewcopies20.tx.1.subsample.1.biom

categorize_by_function.py -i Unotu4hnewcopies20.tx.1.subsample.1.biom -c KEGG_Pathways -l 2 -o Unotu4hnewcopies20.tx.1.subsample.1.L1.biom

biom convert -i Unotu4hnewcopies20.tx.1.subsample.1.L1.biom -o Unotu4hnewcopies20.tx.1.subsample.1.L2json.biom --to-json

biom convert --table-type="OTU table" -i Unotu13hnewcopies20.tx.1.subsample.1.biom -o Unotu13hnewcopies20.tx.1.subsample.1.txt --to-tsv --header-key taxonomy

biom convert -i Unotu13hnewcopies20.tx.1.subsample.1.txt -o Unotu13hnewcopies20.tx.1.subsample.1.1json.biom --table-type="OTU table" --to-json --process-obs-metadata taxonomy

normalize_by_copy_number.py -i Unotu13hnewcopies20.tx.1.subsample.1.1json.biom -o Unotu13hnewcopies20.tx.1.subsample.1.biom

predict_metagenomes.py -i Unotu13hnewcopies20.tx.1.subsample.1.biom -o Unotu13hnewcopies20.tx.1.subsample.1.biom

categorize_by_function.py -i Unotu13hnewcopies20.tx.1.subsample.1.biom -c KEGG_Pathways -l 2 -o Unotu13hnewcopies20.tx.1.subsample.1.L1.biom

biom convert -i Unotu13hnewcopies20.tx.1.subsample.1.L1.biom -o Unotu13hnewcopies20.tx.1.subsample.1.L2json.biom --to-json

biom convert --table-type="OTU table" -i Unotu111hnewcopies20.tx.1.subsample.1.biom -o Unotu111hnewcopies20.tx.1.subsample.1.txt --to-tsv --header-key taxonomy

biom convert -i Unotu111hnewcopies20.tx.1.subsample.1.txt -o Unotu111hnewcopies20.tx.1.subsample.1.1json.biom --table-type="OTU table" --to-json --process-obs-metadata taxonomy

normalize_by_copy_number.py -i Unotu111hnewcopies20.tx.1.subsample.1.1json.biom -o Unotu111hnewcopies20.tx.1.subsample.1.biom

predict_metagenomes.py -i Unotu111hnewcopies20.tx.1.subsample.1.biom -o Unotu111hnewcopies20.tx.1.subsample.1.biom

categorize_by_function.py -i Unotu111hnewcopies20.tx.1.subsample.1.biom -c KEGG_Pathways -l 2 -o Unotu111hnewcopies20.tx.1.subsample.1.L1.biom

biom convert -i Unotu111hnewcopies20.tx.1.subsample.1.L1.biom -o Unotu111hnewcopies20.tx.1.subsample.1.L2json.biom --to-json

biom convert --table-type="OTU table" -i Unotu3hnewcopies20.tx.1.subsample.1.biom -o Unotu3hnewcopies20.tx.1.subsample.1.txt --to-tsv --header-key taxonomy

biom convert -i Unotu3hnewcopies20.tx.1.subsample.1.txt -o Unotu3hnewcopies20.tx.1.subsample.1.1json.biom --table-type="OTU table" --to-json --process-obs-metadata taxonomy

normalize_by_copy_number.py -i Unotu3hnewcopies20.tx.1.subsample.1.1json.biom -o Unotu3hnewcopies20.tx.1.subsample.1.biom

predict_metagenomes.py -i Unotu3hnewcopies20.tx.1.subsample.1.biom -o Unotu3hnewcopies20.tx.1.subsample.1.biom

categorize_by_function.py -i Unotu3hnewcopies20.tx.1.subsample.1.biom -c KEGG_Pathways -l 2 -o Unotu3hnewcopies20.tx.1.subsample.1.L1.biom

biom convert -i Unotu3hnewcopies20.tx.1.subsample.1.L1.biom -o Unotu3hnewcopies20.tx.1.subsample.1.L2json.biom --to-json

biom convert --table-type="OTU table" -i Unotu47hnewcopies20.tx.1.subsample.1.biom -o Unotu47hnewcopies20.tx.1.subsample.1.txt --to-tsv --header-key taxonomy

biom convert -i Unotu47hnewcopies20.tx.1.subsample.1.txt -o Unotu47hnewcopies20.tx.1.subsample.1.1json.biom --table-type="OTU table" --to-json --process-obs-metadata taxonomy

normalize_by_copy_number.py -i Unotu47hnewcopies20.tx.1.subsample.1.1json.biom -o Unotu47hnewcopies20.tx.1.subsample.1.biom

predict_metagenomes.py -i Unotu47hnewcopies20.tx.1.subsample.1.biom -o Unotu47hnewcopies20.tx.1.subsample.1.biom

categorize_by_function.py -i Unotu47hnewcopies20.tx.1.subsample.1.biom -c KEGG_Pathways -l 2 -o Unotu47hnewcopies20.tx.1.subsample.1.L1.biom

biom convert -i Unotu47hnewcopies20.tx.1.subsample.1.L1.biom -o Unotu47hnewcopies20.tx.1.subsample.1.L2json.biom --to-json

biom convert --table-type="OTU table" -i Hotu33hnewcopies20.tx.1.subsample.1.biom -o Hotu33hnewcopies20.tx.1.subsample.1.txt --to-tsv --header-key taxonomy

biom convert -i Hotu33hnewcopies20.tx.1.subsample.1.txt -o Hotu33hnewcopies20.tx.1.subsample.1.1json.biom --table-type="OTU table" --to-json --process-obs-metadata taxonomy

normalize_by_copy_number.py -i Hotu33hnewcopies20.tx.1.subsample.1.1json.biom -o Hotu33hnewcopies20.tx.1.subsample.1.biom

predict_metagenomes.py -i Hotu33hnewcopies20.tx.1.subsample.1.biom -o Hotu33hnewcopies20.tx.1.subsample.1.biom

categorize_by_function.py -i Hotu33hnewcopies20.tx.1.subsample.1.biom -c KEGG_Pathways -l 2 -o Hotu33hnewcopies20.tx.1.subsample.1.L1.biom

biom convert -i Hotu33hnewcopies20.tx.1.subsample.1.L1.biom -o Hotu33hnewcopies20.tx.1.subsample.1.L2json.biom --to-json

biom convert --table-type="OTU table" -i Hotu8hnewcopies20.tx.1.subsample.1.biom -o Hotu8hnewcopies20.tx.1.subsample.1.txt --to-tsv --header-key taxonomy

biom convert -i Hotu8hnewcopies20.tx.1.subsample.1.txt -o Hotu8hnewcopies20.tx.1.subsample.1.1json.biom --table-type="OTU table" --to-json --process-obs-metadata taxonomy

normalize_by_copy_number.py -i Hotu8hnewcopies20.tx.1.subsample.1.1json.biom -o Hotu8hnewcopies20.tx.1.subsample.1.biom

predict_metagenomes.py -i Hotu8hnewcopies20.tx.1.subsample.1.biom -o Hotu8hnewcopies20.tx.1.subsample.1.biom

categorize_by_function.py -i Hotu8hnewcopies20.tx.1.subsample.1.biom -c KEGG_Pathways -l 2 -o Hotu8hnewcopies20.tx.1.subsample.1.L1.biom

biom convert -i Hotu8hnewcopies20.tx.1.subsample.1.L1.biom -o Hotu8hnewcopies20.tx.1.subsample.1.L2json.biom --to-json

biom convert --table-type="OTU table" -i Hotu123hnewcopies20.tx.1.subsample.1.biom -o Hotu123hnewcopies20.tx.1.subsample.1.txt --to-tsv --header-key taxonomy

biom convert -i Hotu123hnewcopies20.tx.1.subsample.1.txt -o Hotu123hnewcopies20.tx.1.subsample.1.1json.biom --table-type="OTU table" --to-json --process-obs-metadata taxonomy

normalize_by_copy_number.py -i Hotu123hnewcopies20.tx.1.subsample.1.1json.biom -o Hotu123hnewcopies20.tx.1.subsample.1.biom

predict_metagenomes.py -i Hotu123hnewcopies20.tx.1.subsample.1.biom -o Hotu123hnewcopies20.tx.1.subsample.1.biom

categorize_by_function.py -i Hotu123hnewcopies20.tx.1.subsample.1.biom -c KEGG_Pathways -l 2 -o Hotu123hnewcopies20.tx.1.subsample.1.L1.biom

biom convert -i Hotu123hnewcopies20.tx.1.subsample.1.L1.biom -o Hotu123hnewcopies20.tx.1.subsample.1.L2json.biom --to-json

biom convert --table-type="OTU table" -i Hotu24hnewcopies20.tx.1.subsample.1.biom -o Hotu24hnewcopies20.tx.1.subsample.1.txt --to-tsv --header-key taxonomy

biom convert -i Hotu24hnewcopies20.tx.1.subsample.1.txt -o Hotu24hnewcopies20.tx.1.subsample.1.1json.biom --table-type="OTU table" --to-json --process-obs-metadata taxonomy

normalize_by_copy_number.py -i Hotu24hnewcopies20.tx.1.subsample.1.1json.biom -o Hotu24hnewcopies20.tx.1.subsample.1.biom

predict_metagenomes.py -i Hotu24hnewcopies20.tx.1.subsample.1.biom -o Hotu24hnewcopies20.tx.1.subsample.1.biom

categorize_by_function.py -i Hotu24hnewcopies20.tx.1.subsample.1.biom -c KEGG_Pathways -l 2 -o Hotu24hnewcopies20.tx.1.subsample.1.L1.biom

biom convert -i Hotu24hnewcopies20.tx.1.subsample.1.L1.biom -o Hotu24hnewcopies20.tx.1.subsample.1.L2json.biom --to-json

biom convert --table-type="OTU table" -i Hotu103hnewcopies20.tx.1.subsample.1.biom -o Hotu103hnewcopies20.tx.1.subsample.1.txt --to-tsv --header-key taxonomy

biom convert -i Hotu103hnewcopies20.tx.1.subsample.1.txt -o Hotu103hnewcopies20.tx.1.subsample.1.1json.biom --table-type="OTU table" --to-json --process-obs-metadata taxonomy

normalize_by_copy_number.py -i Hotu103hnewcopies20.tx.1.subsample.1.1json.biom -o Hotu103hnewcopies20.tx.1.subsample.1.biom

predict_metagenomes.py -i Hotu103hnewcopies20.tx.1.subsample.1.biom -o Hotu103hnewcopies20.tx.1.subsample.1.biom

categorize_by_function.py -i Hotu103hnewcopies20.tx.1.subsample.1.biom -c KEGG_Pathways -l 2 -o Hotu103hnewcopies20.tx.1.subsample.1.L1.biom

biom convert -i Hotu103hnewcopies20.tx.1.subsample.1.L1.biom -o Hotu103hnewcopies20.tx.1.subsample.1.L2json.biom --to-json

biom convert --table-type="OTU table" -i Hotu175hnewcopies20.tx.1.subsample.1.biom -o Hotu175hnewcopies20.tx.1.subsample.1.txt --to-tsv --header-key taxonomy

biom convert -i Hotu175hnewcopies20.tx.1.subsample.1.txt -o Hotu175hnewcopies20.tx.1.subsample.1.1json.biom --table-type="OTU table" --to-json --process-obs-metadata taxonomy

normalize_by_copy_number.py -i Hotu175hnewcopies20.tx.1.subsample.1.1json.biom -o Hotu175hnewcopies20.tx.1.subsample.1.biom

predict_metagenomes.py -i Hotu175hnewcopies20.tx.1.subsample.1.biom -o Hotu175hnewcopies20.tx.1.subsample.1.biom

categorize_by_function.py -i Hotu175hnewcopies20.tx.1.subsample.1.biom -c KEGG_Pathways -l 2 -o Hotu175hnewcopies20.tx.1.subsample.1.L1.biom

biom convert -i Hotu175hnewcopies20.tx.1.subsample.1.L1.biom -o Hotu175hnewcopies20.tx.1.subsample.1.L2json.biom --to-json

biom convert --table-type="OTU table" -i Hotu30hnewcopies20.tx.1.subsample.1.biom -o Hotu30hnewcopies20.tx.1.subsample.1.txt --to-tsv --header-key taxonomy

biom convert -i Hotu30hnewcopies20.tx.1.subsample.1.txt -o Hotu30hnewcopies20.tx.1.subsample.1.1json.biom --table-type="OTU table" --to-json --process-obs-metadata taxonomy

normalize_by_copy_number.py -i Hotu30hnewcopies20.tx.1.subsample.1.1json.biom -o Hotu30hnewcopies20.tx.1.subsample.1.biom

predict_metagenomes.py -i Hotu30hnewcopies20.tx.1.subsample.1.biom -o Hotu30hnewcopies20.tx.1.subsample.1.biom

categorize_by_function.py -i Hotu30hnewcopies20.tx.1.subsample.1.biom -c KEGG_Pathways -l 2 -o Hotu30hnewcopies20.tx.1.subsample.1.L1.biom

biom convert -i Hotu30hnewcopies20.tx.1.subsample.1.L1.biom -o Hotu30hnewcopies20.tx.1.subsample.1.L2json.biom --to-json

biom convert --table-type="OTU table" -i Hotu88hnewcopies20.tx.1.subsample.1.biom -o Hotu88hnewcopies20.tx.1.subsample.1.txt --to-tsv --header-key taxonomy

biom convert -i Hotu88hnewcopies20.tx.1.subsample.1.txt -o Hotu88hnewcopies20.tx.1.subsample.1.1json.biom --table-type="OTU table" --to-json --process-obs-metadata taxonomy

normalize_by_copy_number.py -i Hotu88hnewcopies20.tx.1.subsample.1.1json.biom -o Hotu88hnewcopies20.tx.1.subsample.1.biom

predict_metagenomes.py -i Hotu88hnewcopies20.tx.1.subsample.1.biom -o Hotu88hnewcopies20.tx.1.subsample.1.biom

categorize_by_function.py -i Hotu88hnewcopies20.tx.1.subsample.1.biom -c KEGG_Pathways -l 2 -o Hotu88hnewcopies20.tx.1.subsample.1.L1.biom

biom convert -i Hotu88hnewcopies20.tx.1.subsample.1.L1.biom -o Hotu88hnewcopies20.tx.1.subsample.1.L2json.biom --to-json

biom convert --table-type="OTU table" -i Hotu75hnewcopies20.tx.1.subsample.1.biom -o Hotu75hnewcopies20.tx.1.subsample.1.txt --to-tsv --header-key taxonomy

biom convert -i Hotu75hnewcopies20.tx.1.subsample.1.txt -o Hotu75hnewcopies20.tx.1.subsample.1.1json.biom --table-type="OTU table" --to-json --process-obs-metadata taxonomy

normalize_by_copy_number.py -i Hotu75hnewcopies20.tx.1.subsample.1.1json.biom -o Hotu75hnewcopies20.tx.1.subsample.1.biom

predict_metagenomes.py -i Hotu75hnewcopies20.tx.1.subsample.1.biom -o Hotu75hnewcopies20.tx.1.subsample.1.biom

categorize_by_function.py -i Hotu75hnewcopies20.tx.1.subsample.1.biom -c KEGG_Pathways -l 2 -o Hotu75hnewcopies20.tx.1.subsample.1.L1.biom

biom convert -i Hotu75hnewcopies20.tx.1.subsample.1.L1.biom -o Hotu75hnewcopies20.tx.1.subsample.1.L2json.biom --to-json

biom convert --table-type="OTU table" -i Hotu27hnewcopies20.tx.1.subsample.1.biom -o Hotu27hnewcopies20.tx.1.subsample.1.txt --to-tsv --header-key taxonomy

biom convert -i Hotu27hnewcopies20.tx.1.subsample.1.txt -o Hotu27hnewcopies20.tx.1.subsample.1.1json.biom --table-type="OTU table" --to-json --process-obs-metadata taxonomy

normalize_by_copy_number.py -i Hotu27hnewcopies20.tx.1.subsample.1.1json.biom -o Hotu27hnewcopies20.tx.1.subsample.1.biom

predict_metagenomes.py -i Hotu27hnewcopies20.tx.1.subsample.1.biom -o Hotu27hnewcopies20.tx.1.subsample.1.biom

categorize_by_function.py -i Hotu27hnewcopies20.tx.1.subsample.1.biom -c KEGG_Pathways -l 2 -o Hotu27hnewcopies20.tx.1.subsample.1.L1.biom

biom convert -i Hotu27hnewcopies20.tx.1.subsample.1.L1.biom -o Hotu27hnewcopies20.tx.1.subsample.1.L2json.biom --to-json

biom convert --table-type="OTU table" -i Hotu4hnewcopies20.tx.1.subsample.1.biom -o Hotu4hnewcopies20.tx.1.subsample.1.txt --to-tsv --header-key taxonomy

biom convert -i Hotu4hnewcopies20.tx.1.subsample.1.txt -o Hotu4hnewcopies20.tx.1.subsample.1.1json.biom --table-type="OTU table" --to-json --process-obs-metadata taxonomy

normalize_by_copy_number.py -i Hotu4hnewcopies20.tx.1.subsample.1.1json.biom -o Hotu4hnewcopies20.tx.1.subsample.1.biom

predict_metagenomes.py -i Hotu4hnewcopies20.tx.1.subsample.1.biom -o Hotu4hnewcopies20.tx.1.subsample.1.biom

categorize_by_function.py -i Hotu4hnewcopies20.tx.1.subsample.1.biom -c KEGG_Pathways -l 2 -o Hotu4hnewcopies20.tx.1.subsample.1.L1.biom

biom convert -i Hotu4hnewcopies20.tx.1.subsample.1.L1.biom -o Hotu4hnewcopies20.tx.1.subsample.1.L2json.biom --to-json

biom convert --table-type="OTU table" -i Hotu13hnewcopies20.tx.1.subsample.1.biom -o Hotu13hnewcopies20.tx.1.subsample.1.txt --to-tsv --header-key taxonomy

biom convert -i Hotu13hnewcopies20.tx.1.subsample.1.txt -o Hotu13hnewcopies20.tx.1.subsample.1.1json.biom --table-type="OTU table" --to-json --process-obs-metadata taxonomy

normalize_by_copy_number.py -i Hotu13hnewcopies20.tx.1.subsample.1.1json.biom -o Hotu13hnewcopies20.tx.1.subsample.1.biom

predict_metagenomes.py -i Hotu13hnewcopies20.tx.1.subsample.1.biom -o Hotu13hnewcopies20.tx.1.subsample.1.biom

categorize_by_function.py -i Hotu13hnewcopies20.tx.1.subsample.1.biom -c KEGG_Pathways -l 2 -o Hotu13hnewcopies20.tx.1.subsample.1.L1.biom

biom convert -i Hotu13hnewcopies20.tx.1.subsample.1.L1.biom -o Hotu13hnewcopies20.tx.1.subsample.1.L2json.biom --to-json

biom convert --table-type="OTU table" -i Hotu111hnewcopies20.tx.1.subsample.1.biom -o Hotu111hnewcopies20.tx.1.subsample.1.txt --to-tsv --header-key taxonomy

biom convert -i Hotu111hnewcopies20.tx.1.subsample.1.txt -o Hotu111hnewcopies20.tx.1.subsample.1.1json.biom --table-type="OTU table" --to-json --process-obs-metadata taxonomy

normalize_by_copy_number.py -i Hotu111hnewcopies20.tx.1.subsample.1.1json.biom -o Hotu111hnewcopies20.tx.1.subsample.1.biom

predict_metagenomes.py -i Hotu111hnewcopies20.tx.1.subsample.1.biom -o Hotu111hnewcopies20.tx.1.subsample.1.biom

categorize_by_function.py -i Hotu111hnewcopies20.tx.1.subsample.1.biom -c KEGG_Pathways -l 2 -o Hotu111hnewcopies20.tx.1.subsample.1.L1.biom

biom convert -i Hotu111hnewcopies20.tx.1.subsample.1.L1.biom -o Hotu111hnewcopies20.tx.1.subsample.1.L2json.biom --to-json

biom convert --table-type="OTU table" -i Hotu3hnewcopies20.tx.1.subsample.1.biom -o Hotu3hnewcopies20.tx.1.subsample.1.txt --to-tsv --header-key taxonomy

biom convert -i Hotu3hnewcopies20.tx.1.subsample.1.txt -o Hotu3hnewcopies20.tx.1.subsample.1.1json.biom --table-type="OTU table" --to-json --process-obs-metadata taxonomy

normalize_by_copy_number.py -i Hotu3hnewcopies20.tx.1.subsample.1.1json.biom -o Hotu3hnewcopies20.tx.1.subsample.1.biom

predict_metagenomes.py -i Hotu3hnewcopies20.tx.1.subsample.1.biom -o Hotu3hnewcopies20.tx.1.subsample.1.biom

categorize_by_function.py -i Hotu3hnewcopies20.tx.1.subsample.1.biom -c KEGG_Pathways -l 2 -o Hotu3hnewcopies20.tx.1.subsample.1.L1.biom

biom convert -i Hotu3hnewcopies20.tx.1.subsample.1.L1.biom -o Hotu3hnewcopies20.tx.1.subsample.1.L2json.biom --to-json

biom convert --table-type="OTU table" -i Hotu47hnewcopies20.tx.1.subsample.1.biom -o Hotu47hnewcopies20.tx.1.subsample.1.txt --to-tsv --header-key taxonomy

biom convert -i Hotu47hnewcopies20.tx.1.subsample.1.txt -o Hotu47hnewcopies20.tx.1.subsample.1.1json.biom --table-type="OTU table" --to-json --process-obs-metadata taxonomy

normalize_by_copy_number.py -i Hotu47hnewcopies20.tx.1.subsample.1.1json.biom -o Hotu47hnewcopies20.tx.1.subsample.1.biom

predict_metagenomes.py -i Hotu47hnewcopies20.tx.1.subsample.1.biom -o Hotu47hnewcopies20.tx.1.subsample.1.biom

categorize_by_function.py -i Hotu47hnewcopies20.tx.1.subsample.1.biom -c KEGG_Pathways -l 2 -o Hotu47hnewcopies20.tx.1.subsample.1.L1.biom

biom convert -i Hotu47hnewcopies20.tx.1.subsample.1.L1.biom -o Hotu47hnewcopies20.tx.1.subsample.1.L2json.biom --to-json

**Statistical analysis**

homova(phylip=hnewcopies20.tx.1.subsample.braycurtis.1.lt.dist, design=editcr20healthid.design)

homova(phylip=hnewcopies20.tx.1.subsample.jclass.1.lt.dist, design=editcr20healthid.design)

homova(phylip=hnewcopies20.tx.1.subsample.lennon.1.lt.dist, design=editcr20healthid.design)

homova(phylip=hnewcopies20.tx.1.subsample.morisitahorn.1.lt.dist, design=editcr20healthid.design)

homova(phylip=hnewcopies20.tx.1.subsample.sorabund.1.lt.dist, design=editcr20healthid.design)

homova(phylip=hnewcopies20.tx.1.subsample.thetan.1.lt.dist, design=editcr20healthid.design)

homova(phylip=hnewcopies20.tx.1.subsample.thetayc.1.lt.dist, design=editcr20healthid.design)

amova(phylip=edith20.tx.1.subsample.braycurtis.1.lt.dist, design=20health.design)

amova(phylip=edith20.tx.1.subsample.jclass.1.lt.dist, design=20health.design)

amova(phylip=edith20.tx.1.subsample.lennon.1.lt.dist, design=20health.design)

amova(phylip=edith20.tx.1.subsample.morisitahorn.1.lt.dist, design=20health.design)

amova(phylip=edith20.tx.1.subsample.sorabund.1.lt.dist, design=20health.design)

amova(phylip=edith20.tx.1.subsample.thetan.1.lt.dist, design=20health.design)

amova(phylip=edith20.tx.1.subsample.thetayc.1.lt.dist, design=20health.design)
